# Salt stress and senescence: identification of cross-talk regulatory components

**DOI:** 10.1093/jxb/eru173

**Published:** 2014-05-06

**Authors:** Annapurna Devi Allu, Aleksandra Maria Soja, Anhui Wu, Jedrzej Szymanski, Salma Balazadeh

**Affiliations:** ^1^University of Potsdam, Institute of Biochemistry and Biology, Karl-Liebknecht-Straße 24–25, Haus 20, D-14476 Potsdam-Golm, Germany; ^2^Max-Planck Institute of Molecular Plant Physiology, Plant Signaling Group, Am Mühlenberg 1, D-14476 Potsdam-Golm, Germany; ^3^Max-Planck Institute of Molecular Plant Physiology, Department of Molecular Physiology, Am Mühlenberg 1, D-14476 Potsdam-Golm, Germany

**Keywords:** *Arabidopsis*, hydrogen peroxide, longevity, reactive oxygen species, salt stress, senescence, signal cross-talk, transcription factor.

## Abstract

Leaf senescence is an active process with a pivotal impact on plant productivity. It results from extensive signalling cross-talk coordinating environmental factors with intrinsic age-related mechanisms. Although many studies have shown that leaf senescence is affected by a range of external parameters, knowledge about the regulatory systems that govern the interplay between developmental programmes and environmental stress is still vague. Salinity is one of the most important environmental stresses that promote leaf senescence and thus affect crop yield. Improving salt tolerance by avoiding or delaying senescence under stress will therefore play an important role in maintaining high agricultural productivity. Experimental evidence suggests that hydrogen peroxide (H_2_O_2_) functions as a common signalling molecule in both developmental and salt-induced leaf senescence. In this study, microarray-based gene expression profiling on *Arabidopsis thaliana* plants subjected to long-term salinity stress to induce leaf senescence was performed, together with co-expression network analysis for H_2_O_2_-responsive genes that are mutually up-regulated by salt induced- and developmental leaf senescence. Promoter analysis of tightly co-expressed genes led to the identification of seven *cis*-regulatory motifs, three of which were known previously, namely CACGTGT and AAGTCAA, which are associated with reactive oxygen species (ROS)-responsive genes, and CCGCGT, described as a stress-responsive regulatory motif, while the others, namely ACGCGGT, AGCMGNC, GMCACGT, and TCSTYGACG were not characterized previously. These motifs are proposed to be novel elements involved in the H_2_O_2_-mediated control of gene expression during salinity stress-triggered and developmental senescence, acting through upstream transcription factors that bind to these sites.

## Summary

Long-term salinity stress induces senescence, probably through an involvement of hydrogen peroxide (H_2_O_2_)-mediated signalling. This study identifies candidate H_2_O_2_-responsive *cis*-regulatory elements governing gene expression during salinity stress-triggered and developmental senescence.

## Introduction

During the course of senescence, nutrients accumulated in the growing and maturing leaves are exported to actively growing organs such as young leaves, developing fruits, or flowers and storage organs. This mobilization is continued until most of the nutrients are removed from the senescing leaves (Hörtensteiner and Feller, 2002). Therefore, leaf senescence is a critical developmental process for plant fitness. Global transcriptome studies of senescent *Arabidopsis thaliana* leaves indicated that differential gene expression plays an important role in the regulation of this process (Buchanan-Wollaston *et al.*, 2005; van der Graaff *et al*., 2006). Identifying and understanding the functions of the genes that initiate and carry out senescence are essential to manipulate senescence for economic purposes (e.g. to increase biomass and crop yield, and modify production traits).

Developmental senescence is a highly regulated genetically controlled degenerative process. As senescence can normally not be induced easily in young leaves of *Arabidopsis*, it was proposed that age-related changes (ARCs) are needed to allow the execution of senescence at a later developmental stage of a leaf (Jibran *et al.*, 2013). Although the molecular or cellular nature of age-related factors that manifest ARCs are not well defined at present, the termination of cell division or the end of leaf cell expansion may represent such factors (discussed in Jibran *et al.*, 2013). Also, ARCs occurring at the level of various phytohormones (i.e. ethylene, cytokinins, jasmonic acid, and salicylic acid), metabolites (such as sugars), and active forms of oxygen [reactive oxygen species (ROS)] may contribute to establish a senescence-competent status of the leaf (Guo and Gan, 2005).

Of the various possible inducers of the senescence programme, ROS have attracted a lot of attention during the past few years. In addition to their harmful effect on cellular components, ROS are now recognized as an essential component of many developmental and physiological processes including flowering, senescence, and root development (Mittler *et al.*, 2004; Zimmermann and Zentgraf, 2005; Zimmermann *et al.*, 2006; Tsukagoshi *et al.*, 2010). As by-products of aerobic metabolism, ROS are continuously produced in different cellular compartments and their endogenous levels are controlled in a finely tuned manner by a series of enzymatic and non-enzymatic antioxidants. It is becoming increasingly evident that the loss of certain antioxidant activities and the subsequent accumulation of ROS [superoxide, singlet oxygen, hydroxyl radical, and hydrogen peroxide (H_2_O_2_)] with age are signals for the initiation of leaf senescence (Leshem, 1988; Navabpour *et al.*, 2003; Mittler *et al.*, 2004; Zimmermann and Zentgraf, 2005). However, currently their specific roles in the regulation of the senescence process are far from being understood. Transcript profiling experiments revealed massive changes in gene expression in response to various ROS-generating conditions, including to H_2_O_2_ treatment itself (Vandenabeele *et al.*, 2003; Vanderauwera *et al.*, 2005; Balazadeh *et al.*, 2012). This includes expression changes of a large number of senescence-associated transcription factors, further supporting the involvement of ROS in the genetically controlled process of ageing.

Although leaf senescence is largely governed by a genetic programme, abiotic stresses including nutrient (e.g. nitrogen) deprivation, extended darkness, drought, cold, high temperature, salt stress, or wounding are well known to affect the initiation and progression of senescence (Whitehead *et al.*, 1984; Becker and Apel, 1993; Lutts *et al.*, 1996; Yoshida, 2003; Munné-Bosch and Alegre, 2004; Buchanan-Wollaston *et al.*, 2005; Munns, 2005; Parlitz *et al.*, 2011). Under natural conditions, plants are continuously exposed to multiple stresses, and as non-motile organisms they must adjust their physiology to an ever-changing surrounding environment for survival. An example for such an adjustment is the acceleration of vegetative growth, combined with the promotion of leaf senescence, to enter rapidly into the reproductive phase and reach the next generation. Such stress-modulated developmental changes require a sophisticated integration of regulatory networks of abiotic stress responses with the developmental senescence programme.

Data from large-scale expression profiling experiments obtained from plants undergoing developmental or abiotic stress-induced senescence (Buchanan-Wollaston *et al.*, 2005; van der Graaff *et al*., 2006; Parlitz *et al.*, 2011) indicate an overlap in the expression changes and suggest the existence of signalling cross-talk linking the different types of senescence. In addition, various late-flowering and/or stay-green mutants in *Arabidopsis* such as *gigantea* (*gi*), *oresara1* (*ore1*), *ore3*, and *ore9* indicate a possible link between longevity and stress tolerance (Koornneef *et al.*, 1991; Oh *et al.*, 1997; Kurepa *et al.*, 1998; Park *et al.*, 1999; Woo *et al.*, 2004). In *Arabidopsis*, the *anac092-1* stay-green mutant retained chlorophyll at a higher level than the wild type when salt stressed for several days (150mM NaCl), indicating a role for the NAC transcription factor ANAC092/ORE1 not only in developmental senescence (Kim *et al.*, 2009), but also in the regulation of salt-promoted senescence (Balazadeh *et al.*, 2010). Overexpression of *JUB1*, a further member of the NAC transcription factor gene family, in transgenic *Arabidopsis* extends plant longevity and confers abiotic stress tolerance through a tight regulation of the cellular H_2_O_2_ level (Wu *et al.*, 2012). OsTZF1, a member of the CCCH-type zinc-finger gene family in rice (*Oryza sativa*), is a negative regulator of developmental and abiotic stress-induced senescence. Transgenic plants overexpressing *OsTZF1* exhibit delayed senescence under various abiotic stress conditions including high salinity, darkness, and dehydration, indicating that both tolerance to oxidative stress and retarded senescence are based on the same cellular factor(s) (Jan *et al.*, 2013). These and other reports support the model that stress-induced and senescence regulatory pathways share common elements.

Salinity stress is a major abiotic stress limiting plant growth and productivity worldwide. By triggering a wide range of cellular events, salinity, like other abiotic stresses, superimposes its downstream effects on the existing developmental signalling processes. This leads to the activation of whole-plant responses, such as growth reduction, changes in biomass allocation, leaf senescence, and death of plants (Volkmar *et al.*, 1998; Munns, 2002; Pic *et al.*, 2002; Munné-Bosch and Alegre, 2004). It has been proposed that many salt stress-triggered processes, such as a decline in photosynthetic activity or an increase in membrane damage, reflect a hastening of the naturally occurring senescence process (Dwidedi *et al.*, 1979; Dhindsa *et al.*, 1981). In sweet potato, treatment of detached mature leaves with NaCl (140mM and 210mM) accelerated leaf senescence in a dose-dependent manner on days 6 and 9 after treatment. The early leaf senescence induced by salt was accompanied by a decrease in chlorophyll content, reduction of photosynthetic efficiency (*F*_v_/*F*_m_), and an elevation of H_2_O_2_ level (Chen *et al.*, 2012). The fact that ROS, especially H_2_O_2_, accumulate during both salinity stress (Gomez *et al.*, 2004; Rubio *et al.*, 2009; Hanqing *et al.*, 2010; Chen *et al.*, 2012) and developmental senescence (Zimmermann and Zentgraf, 2005; Bieker *et al.*, 2012) suggests the existence of ROS-mediated cross-talk between the two processes.

Although salinity triggers leaf senescence in different plant species, the regulatory mechanisms integrating salt stress signalling with senescence are incompletely known at present. With respect to agriculture, however, a better understanding of such processes is expected to aid in the breeding of crops with improved salt tolerance by avoiding or delaying senescence during salinity stress. Although in *Arabidopsis* various transcriptome studies were performed in the past to characterize the global expressional responses to salt stress (Kreps *et al*., 2002; Seki *et al.*, 2002; Taji *et al.*, 2004; Gong *et al.*, 2005; Matsui *et al.*, 2008; Zeller *et al.*, 2009), little attention has so far been paid to studying the regulation of gene expression during senescence in salt-stressed plants. Here, the transcriptomes of *Arabidopsis* leaves during salt-induced senescence were therefore analysed and compared with transcriptomes from leaves during developmental senescence (Buchanan-Wollaston *et al.*, 2005; Balazadeh *et al.*, 2008; Breeze *et al.*, 2011) and plants subjected to H_2_O_2_ treatment. By integrating co-expression data with promoter analyses, candidate *cis*-regulatory elements (CREs) governing gene expression under the three conditions examined could be identified. The results thus provide novel information relevant for studies on the transcriptional programmes that control H_2_O_2_-mediated salt-induced senescence and shed light on the complex signal transduction pathways that regulate leaf senescence under salt stress.

## Materials and methods

### General

Standard molecular techniques were performed as described (Sambrook and Russell, 2001). Oligonucleotides were obtained from Eurofins MWG Operon (Ebersberg, Germany). The Arabidopsis Hormone Database (http://ahd.cbi.pku.edu.cn/) and the Arabidopsis eFP Browser (http://bar.utoronto.ca/efp/cgi-bin/efpWeb.cgi) were used for expression analyses.

### Plant material and stress treatments

*Arabidopsis thaliana* (L.) Heynh. (Col-0 ecotype) was germinated and grown on 0.5× Murashige and Skoog (MS) agar medium containing 1% sucrose. The plants were grown in a growth chamber at 22 °C under a 16h day (140 μmol m^–2^ s^–1^)/8h night regime. For H_2_O_2_ treatment, 2-week-old seedlings were removed from agar and transferred to flasks containing liquid MS medium (1% sucrose) and subjected to 10mM H_2_O_2_ for the indicated times.

For salinity stress treatment, *Arabidopsis* seeds were surface sterilized. Plants were grown in a hydroponic system in a climate chamber at an 8h light (150 μmol m^–2^ s^–1^ 20 °C, 60% relative humidity)/16h dark (16 °C, 75% relative humidity) cycle; the light period stared at 06:00h. Seeds were sown on glass wool placed on a plastic tray having small holes in its bottom, which allows the contact of the glass wool and later the roots with the liquid medium in a plastic box placed below. The medium used was essentially as described by Loque *et al.* (2003). Six plants were grown in each hydroponic box and the growth medium was replaced with fresh, autoclaved solution every seventh day to limit the growth of microorganisms and supply enough nutrients for normal plant growth. Stress treatments were initiated at 10:00h by adding NaCl (final concentration: 150mM) to the liquid medium, and medium without NaCl served as control. Leaves (entire shoots) of treated and control plants were frozen in liquid nitrogen and used for expression profiling. The experiment was performed in three independent biological replications.

### Fluorescence measurement using pulse amplitude modulation (PAM)

Maximal photosynthetic efficiency of photosystem II (PSII; *F*_v_/*F*_m_) was measured using a PAM 2000 fluorometer (Heinz Walz, Effeltrich, Germany). Rosette leaves were dark-adapted for 5min and *F*_v_/*F*_m_ was measured at 20 °C. The 5min dark treatment resulted in the complete oxidation of Q_A_.

### Measurement of chlorophyll content

Chlorophyll content was determined using a SPAD analyser (N-tester, Hydro Agri Immingham, UK).

### Expression analysis by qRT-PCR

Total RNA extraction, cDNA synthesis, and quantitative real-time PCR (qRT-PCR) were carried out as described (Caldana *et al.*, 2007; Balazadeh *et al.*, 2008). *ACTIN2* (*At3g18780*) served as reference gene in all qRT-PCR experiments. *ACTIN2* primers were Actin2-fwd (5′-TCCCTCAGCACATTCCAGCAGAT-3′) and Actin2-rev (5′-AACGATTCCTGGACCTGCCTCATC-3′). *SAG12* and *WRKY53* primer sequences were as follows: SAG12-fwd, 5′-ACAAAGGCGAAGACGCTACTTG-3′ and SAG12-rev, 5′-ACC GGGACATCCTCATAACCTG-3′; WRKY53-fwd, 5′-ATCCC GGCAGTGTTCCAGAATC-3′ and WRKY53-rev, 5′-AGAACC TCCTCCATCGGCAAAC-3′.

### Microarray hybridizations

Affymetrix ATH1 hybridizations were performed by Atlas Biolabs (http://www.atlas-biolabs.com/). Expression data were analysed using Bioconductor (Gentleman *et al.*, 2004). Data quality was evaluated by affy and affyPL packages. Data were normalized with robust multiarray averaging (Irizarry *et al.*, 2003). Statistical testing for differential expression was performed using the Limma Bioconductor package

Expression data were submitted to the NCBI Gene Expression Omnibus (GEO) repository (http://www.ncbi.nlm.nih.gov/geo/) under accession number GSE53308.

### Response classification

Response classification was based on gene differential expression (2-fold expression difference as cut-off) upon H_2_O_2_ (1h and/or 5h 10mM H_2_O_2_) treatment or long-term NaCl treatment (4 d, 150mM NaCl). Developmental senescence-up-regulated genes (SAGs) were extracted from Buchanan-Wollaston *et al.* (2005), Balazadeh *et al.* (2008), and Breeze *et al.* (2011), and developmental senescence-down-regulated genes (SDGs) were extracted from Buchanan-Wollaston *et al.* (2005) and Breeze *et al.* (2011). Up- or down-regulated genes or those exhibiting no response were attributed as having a value of 1, –1, or 0, respectively. In this way, each gene was categorized into one of 26 clusters, each representing one of 26 possible patterns of expression in the three listed conditions.

### Co-expression analysis

Time series data were obtained from NASC (experiments 143 and 140 from the AtGenExpress consortium for oxidative stress and salt stress responses, respectively) and Breeze *et al.* (2011) (developmental senescence). The significance of the differential gene expression in the time series experiments was estimated using paired *t*-test between treatment and the corresponding control. Data sets of 350 genes (cluster 26) identified as robustly up-regulated in all three conditions were extracted. The time series data were Z-transformed independently, centring expression of each gene to 0 and normalizing its standard deviation to 1. Subsequently, three correlation matrices were obtained, one for each data set. Each correlation matrix was transformed into a binary adjacency matrix, using the chosen significance cut-off, and represented as a network. Finally, the intersection of these three networks was obtained, representing the gene co-expression structure conserved across all three experimental conditions. In the intersection network, communities were identified using the greedy search algorithm (Clauset *et al.*, 2004). The robustness of the community search was checked for correlation thresholds 0.1–0.9, and the seven regulatory modules presented herein are stable between the thresholds 0.4 and 0.65. The value applied here (i.e. 0.6) represents a compromise between correlation significance and the size of identified communities.

### *Cis*-regulatory elements analysis

In the approach used here motifs of length 6–12 nucleotides were looked for in the 500bp upstream regions (obtained from http://arabidopsis.org/tools/bulk/sequences/index.jsp). To detect motifs, the ZOOPS model (Bailey and Elkan, 1995) was used, which considers that the motif occurrence can be zero or one in a sequence. The maximum number of sites to find was set to 10. To verify if the identified motifs are previously characterized *Arabidopsis* CREs, the TOMTOM tool (Motif Comparison Tool) was used. TOMTOM searches motif–motif databases and measures statistical similarity between motifs (Gupta *et al.*, 2007). Finally, PatMatch (Yan *et al.*, 2005) (http://www.arabidopsis.org/cgi-bin/patmatch/nph-patmatch.pl) was used to find all genes in the *Arabidopsis* genome containing the given motif in their promoter sequence.

### Function enrichment analysis

agriGO (http://bioinfo.cau.edu.cn/agriGO/index.php) was employed to extract function annotations for genes harbouring a given motif in their promoter sequence. In the present analysis the tool SEA (singular enrichment analysis) was used. SEA determines gene ontology (GO) term enrichment in one group of genes by comparing it with a reference group of genes (Du *et al.*, 2010). As a background, *Arabidopsis* gene models from TAIR9 were used.

## Results

### Salinity induces leaf senescence in plants subjected to long-term moderate stress

**Fig. 1.**
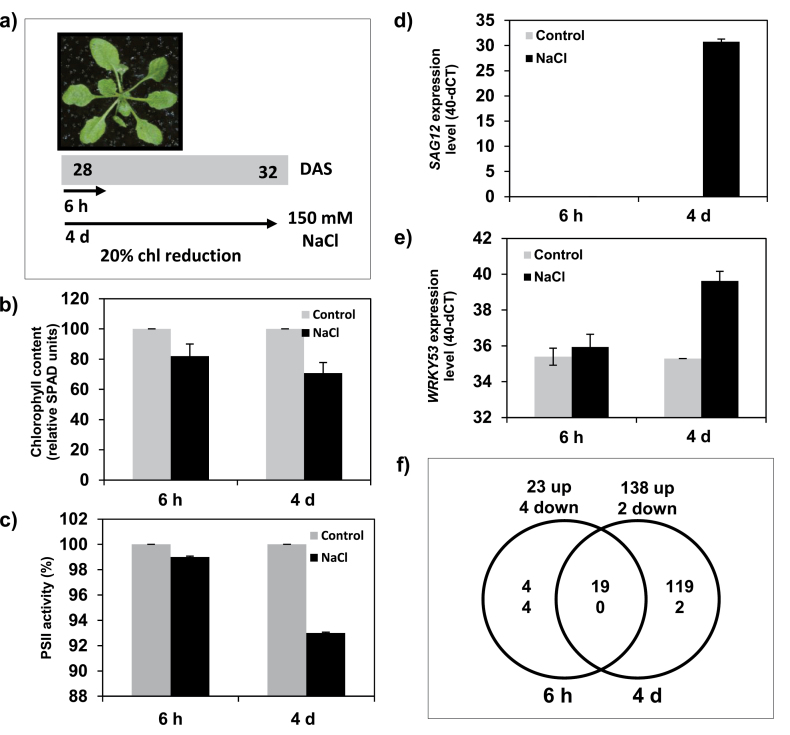
Molecular and physiological analysis of salt-treated plants. (a) Design of the experiment and plant stage selected for salt stress (150mM NaCl) treatment (28 d after sowing, DAS). Samples were collected 6h after the onset of the stress (short-term salinity stress). The later time point (4 d after start of salinity stress) was selected based on the percentage reduction of relative leaf chlorophyll (chl) content. (b) Leaf chlorophyll content. (c) Photochemical efficiency of PSII (*F*_v_/*F*_m_). The values in (b) and (c) represent means of data obtained for 13 plants at each data point ±SD. (d) Quantitative RT-PCR analysis of *SAG12* and (e) *WRKY53* expression under control (grey column) and salt stress (black column) conditions at two examined time points. Data are means of three independent experiments ±SD. (f) Venn diagram showing an overview of changes in gene expression (>2-fold) of ROS-responsive genes in 6h and 4 d NaCl-treated samples and their shared responses. The numbers in the Venn diagram indicate the number of genes (upper values indicate the number of up-regulated genes, and lower values indicate the number of down-regulated genes).

In order to study the molecular mechanism of salt-induced senescence and its potential cross-talk with developmental senescence, an experimental condition was first set up under which salinity stress induces characteristics of developmental senescence. To this end, *Arabidopsis* plants were grown hydroponically, allowing sampling of shoots and roots separately. Twenty-eight-day-old *Arabidopsis* plants were salinity stressed by treatment of roots with NaCl (150mM) for 6h and 4 d, respectively (see the Materials and methods). The 6h salt stress did not induce leaf senescence; no change in chlorophyll content and no visible yellowing occurred. Likewise, expression of *SAG12* and *WRKY53* (known senescence marker genes; Noh and Amasino, 1999; Zentgraf *et al.*, 2010) was unaffected in leaves upon short-term salt stress (Fig. 1). In contrast, long-term salt stress (4 d) was accompanied by a reduction in photosynthetic efficiency of PSII and chlorophyll level. The *F*_v_/*F*_m_ ratio and chlorophyll content declined by ~10% and ~20%, respectively, after 4 d of stress, and expression of *SAG12* and *WRKY53* was induced (Fig. 1). These results indicate that leaf senescence in *Arabidopsis* is triggered by salt treatment after 4 d under the experimental conditions used here. Next, the expression level of 179 ROS-responsive genes was tested using a previously established qRT-PCR platform (Wu *et al.*, 2012). Expression profiling was performed in three biological replicates. Considering a 2-fold expression difference as cut-off, a total of 23 and 138 ROS-responsive genes were up-regulated after 6h and 4 d of NaCl treatment, respectively (Fig. 1f; Supplementary Table S1 available at *JXB* online), indicating an increased accumulation of ROS after long-term salt stress.

### Comparison of developmental and salt-triggered senescence transcriptomes

**Fig. 2.**
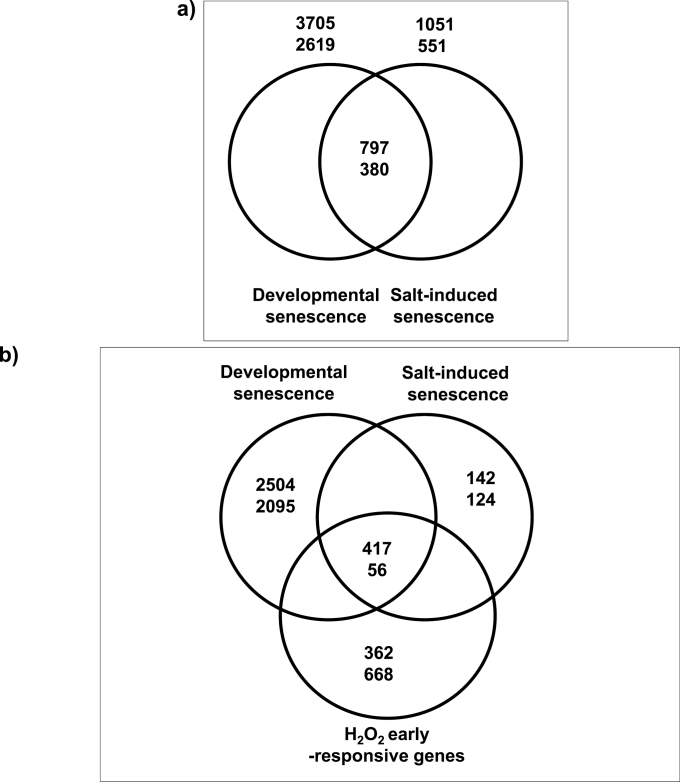
Shared gene expression responses. (a) Venn diagram showing the overlap of genes differentially expressed during developmental and salt-induced senescence. (b) Venn diagram showing the overlap of genes affected during developmental senescence, salt-induced senescence, and upon hydrogen peroxide (H_2_O_2_) treatment. Numbers indicate the number of genes up-regulated (upper values) or down-regulated (lower values) in the different conditions.

In order to identify possible cross-talk components shared between developmental and salt-triggered senescence, the expression profile of *Arabidopsis* leaves was first obtained under the condition of salt-induced senescence (4 d) and then it was compared with the transcriptome of developmental leaf senescence. Genes undergoing expression changes during developmental senescence were previously reported (e.g. Buchanan-Wollaston *et al.*, 2005; Balazadeh *et al.*, 2008; Breeze *et al.*, 2011); using the reported transcriptome data, a list of 3705 developmental senescence-up-regulated and 2619 down-regulated genes was compiled, which was used here for comparison. The analysis revealed genes that are unique to either developmental or salt stress-induced senescence, and those that are shared among the two types of senescence (Fig. 2a; Supplementary Tables S2, S3 at *JXB* online). In total, 1602 genes were differentially expressed after 4 d of NaCl treatment, compared with non-stressed control plants, of which 1051 were up- and 551 were down-regulated (Supplementary Table S2). The majority of the responding genes, namely 797 of the 1051 salinity-up-regulated genes (~76%) and 380 of the 551 down-regulated genes (~69%), are known developmental senescence-up- and down-regulated genes, respectively (χ^2^ test *P*-value <2.2e-16; Fig. 2a; Supplementary Table S3). Such an extensive overlap between the two types of transcriptomes (1177 genes in total) further supports the conclusion that long-term salt treatment of hydroponically grown plants induces a condition similar to that of developmental senescence.

However, expression of 266 genes was specifically altered (142 genes up- and 124 genes down-regulated) in salinity-induced senescence (Supplementary Table S2 at *JXB* online). While the down-regulated genes did not exhibit enrichment in any functional category, the up-regulated genes were enriched in those responsive to salt stress (seven genes), water deprivation (five genes), and abscisic acid (ABA) stimulus (six genes) (Supplementary Table S2). Common for these three groups are the two *LOW-TEMPERATURE-INDUCED* (*LTI*) genes *LTI65* (identical to *RD29B*) and *LTI78* (*RD29A*) (Nordin *et al.*, 1993; Msanne *et al.*, 2011). The most enriched functional group is ‘lipid transport’, represented by five lipid transfer proteins: one LTP type 3 (AT4G33550), three LTP type 5 (AT2G37870, AT3G22620, and AT1G62790), and one LTP type 4 protein (AT3G53980). LTPs are glycosylphosphatidylinositol (GPI)-anchored membrane proteins involved in fatty acid transport during cuticular wax and suberin synthesis (Borner *et al.*, 2003; Debono *et al*., 2009), and some of them have specific functions during flower and embryo development (Pagnussat *et al.*, 2005; Schmid *et al.*, 2005). While none of the LTPs identified in our study has been experimentally characterized, up-regulation of five members of the same gene family indicates regulation of extracellular lipid synthesis during salt-induced senescence. Notably, none of these genes is up-regulated upon short-term (6h) salinity stress (data not shown).

### H_2_O_2_, a potential signalling element in the cross-talk between developmental and salt-triggered senescence

The cellular level of ROS, particularly H_2_O_2_, increases upon salinity stress and when leaves age, suggesting H_2_O_2_ as a potential signalling molecule in both processes (Gomez *et al.*, 2004; Bhattacharjee, 2005; Zimmermann and Zentgraf, 2005; Zimmermann *et al.*, 2006; Rubio *et al.*, 2009; Hanqing *et al.*, 2010; Chen *et al.*, 2012; Bieker *et al.*, 2012). To gain more insight into the possible role of H_2_O_2_ as a cross-talk component of signalling pathways controlling developmental and stress-induced senescence, H_2_O_2_ early-responsive genes were first identified and compared with the senescence transcriptomes. To this end, *Arabidopsis* seedlings were treated with H_2_O_2_ for 1h and 5h, respectively, and subjected to expression profiling using Affymetrix ATH1 arrays. In total, 2228 genes were differentially expressed upon H_2_O_2_ treatment (1214 up and 1014 down) (Supplementary Table S4 at *JXB* online), of which 1030 genes (362 up- and 668 down-regulated) were specifically altered upon H_2_O_2_ treatment, but not during salinity-induced or developmental senescence (Supplementary Table S4). Approximately 40% (473 of 1177) of the genes regulated during both developmental and salt-induced senescence were early H_2_O_2_-responsive (Fig. 2b). Such an extensive overlap further suggests ROS (H_2_O_2_) as a regulator of cross-talk between developmental and salt-induced senescence signalling pathways.

### Identification of regulatory components across experimental conditions

To identify regulatory elements representing cross-talk points between salt-induced and developmental senescence pathways signalled by H_2_O_2,_ the co-expression of genes and the distribution of the CREs in promoters of the genes undergoing significant changes of expression in the three experimental conditions (salt-induced senescence, developmental senescence, and H_2_O_2_ treatment) were explored. A three-step analysis was performed, as outlined in detail below: (i) a set of candidate genes responsive in all three experimental conditions was identified; (ii) putative CREs significantly enriched and common for genes involved in salt-induced senescence, developmental senescence, and oxidative stress responses were identified; and (iii) using correlation analysis, the promoter analysis was then integrated with the co-expression information, highlighting CREs most likely to be responsible for governing the changes in gene expression.

### Gene clustering

Typically, gene clustering is based on distance matrices, such as Euclidean distance or Pearson’s correlation coefficient (Usadel *et al.*, 2009). However, due to the large number of genes and low number of experimental points, a different approach was taken here. Thus, genes were first classified as ‘up-regulated’, ‘down-regulated’, or ‘not affected’ during developmental senescence, salt-induced senescence, or treatment with H_2_O_2_ (1h and/or 5h) and in this way each gene was assigned to one of 26 clusters (Supplementary Table S5 at *JXB* online), each representing one of 26 possible patterns of expression in the three experimental conditions. In the following, a significant change of expression is referred to as gene activation (positive or negative for up- and down-regulation, respectively) in particular experimental set-ups. For example, cluster 14 includes 1704 genes that are positively activated only during developmental senescence, while cluster 26 includes 350 genes positively activated in all three experimental conditions. Importantly, as similarities of gene expression patterns were not looked at specifically, but rather response specificity, each of the 26 clusters includes genes likely to differ remarkably in the timing of the response, its scale, and duration. Thus, as is shown below, within each cluster multiple putative regulatory mechanisms can be identified, responsible for triggering different sets of genes at different times of the response and with different strengths.

To identify CREs potentially involved in H_2_O_2_-mediated salt-induced senescence, only clusters containing genes responsive to all three experimental conditions were selected for promoter motif analysis (clusters 1 and 26).

### Promoter analysis and identification of *cis*-regulatory elements

**Fig. 3.**
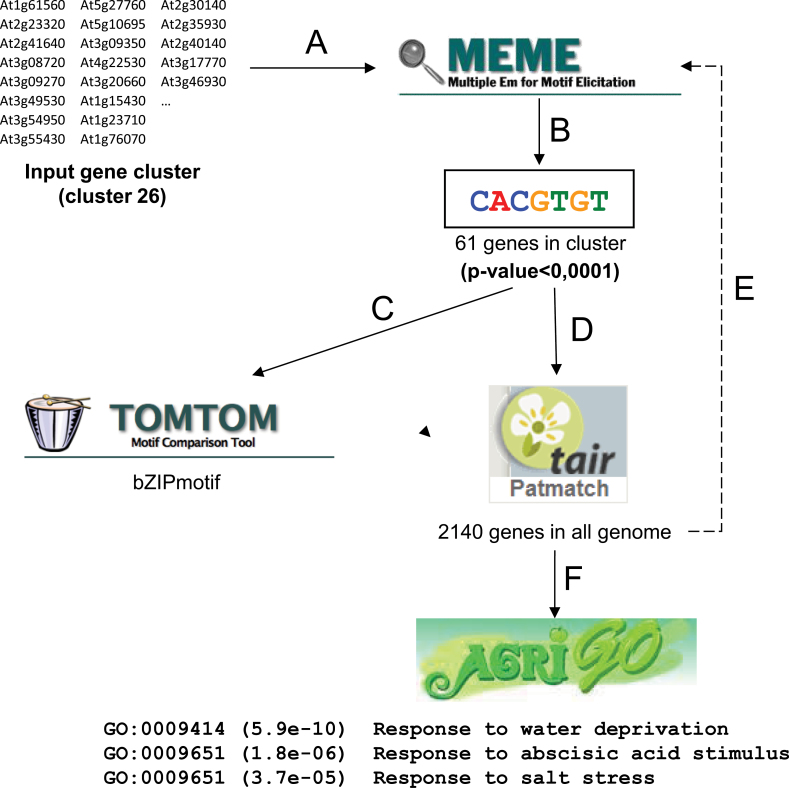
*Cis*-regulatory search pipeline. The analysis pipeline included the following steps. (A) Upstream sequences (500bp) of input genes were retrieved from the TAIR database. (B) MEME Suite was used to identify significantly enriched sequence motifs in the set of queried promoters (here, the CACGTGT motif is given as an example). (C) Candidate motifs were compared with a database of known *cis*-regulatory motifs using TOMTOM. (D) A list of all *Arabidopsis* genes containing the motif was retrieved using PatMatch. (E) This gene set was trimmed to include only those genes that were identified in a repeated MEME analysis. (F) Finally, function enrichment analysis was performed using agriGO. Database logos were taken from the respective web pages.

Sequences of promoters from genes included in clusters 1 (containing 51 genes negatively activated in all three conditions) and 26 (containing 350 genes positively activated in all three conditions) were queried for the presence of CREs using a pipeline of motif search and motif validation tools. To this end, a set of tools available in the MEME Suite (multiple expectation maximization for motif elicitation) (Bailey and Elkan, 1994) and PatMatch (Yan *et al.*, 2005), previously proven successful in multiple similar studies, such as for the prediction of conserved motifs in potato (*Solanum tuberosum* L.) NAC genes (Singh *et al.*, 2013), was used. The analysis pipeline consists of four steps: (i) MEME is used for primary motif search; (ii) TOMTOM defines whether the motif is an already known CRE; (iii) PatMatch determines how many genes in the whole *Arabidopsis* genome contain the motif in the promoter region; and (iv) agriGO reports the functional assignment (Du *et al.*, 2010). Figure 3 shows an example of the identification of a significantly enriched motif (CACGTGT) among the genes exhibiting positive activation in all experimental conditions (cluster 26), which by TOMTOM was identified as an elongated G-box (CACGTG), a bZIP transcription factor recognition site. In parallel, PatMatch detected the presence of the motif in a total of 2140 genes in the *Arabidopsis* genome. Subsequently, to validate the result, all these genes were again used as a query in MEME. Finally, functional analysis by agriGO revealed a significant enrichment of genes containing the motif for multiple stress-related functional categories, including, for example, ‘water deprivation’, ‘abscisic acid stimulus’, and ‘salt stress response’. Note that the functional enrichment analysis is performed on all genes containing the motif of interest, not only those included in the cluster analysed. This is important, since in this way it is shown that the candidate CREs are significantly related to certain gene functions throughout the whole genome and not only within the frame of the identified cluster.

In total, seven motifs significantly enriched only among genes commonly up-regulated upon H_2_O_2_ treatment, and salt-induced and developmental senescence (genes in cluster 26) were found. These are: CACGTGT, AAGTCAA, ACGCGGT, AGCMGNC, GMCACGT, TCSTYGACG, and CCGCGT (Table 1). Three of them (CCGCGT, CACGTGT, and AAGTCAA) are known CREs present in promoters of stress- and ROS-responsive genes (Ma *et al.*, 2012; Petrov *et al.*, 2012); the remaining four are novel and as yet uncharacterized in terms of biological function. Function enrichment analysis indicated that genes harbouring AAGTCAA, CACGTGT, and GMCACGT in their promoters are associated with specific molecular functions, biological processes, or cellular compartments (see below and Supplementary Table S6 at *JXB* online). The same analysis performed on cluster 1 (51 genes down-regulated in all experimental conditions) resulted in the identification of three conserved motifs; however, none of them exhibited a significant enrichment in the clusters analysed or could be associated with a certain biological function.

**Table 1.** Enrichment of the top seven motifs in genes commonly up-regulated upon H_2_O_2_ treatment, and salt-induced and developmental senescence (genes within cluster 26)

	Genes in cluster with motif	Genes outside cluster with motif	Genes in cluster without motif	Remaining genes in input (5999 genes)	*P*-value
AAGTCAA	87	948	263	4701	1.31E-04
CACGTGT	61	548	289	5101	1.17E-05
TCSTYGACG	9	36	341	5613	0.00096247
CCGCGT	32	227	318	5422	3.64E-05
ACGCGGT	19	70	331	5579	5.78E-07
AGCMGNC	61	592	289	5057	9.82E-05
GMCACGT	67	821	283	4828	0.01322002

### Functional enrichment analysis of candidate *cis*-regulatory elements

The present analysis showed that the CACGTGT bZIP (or ACTG ABRE) CRE is very significantly over-represented among genes up-regulated in response to all applied experimental conditions (61 genes, *P*-value <0.00001, calculated by Fischer’s exact test). Interestingly, 56 of these 61 genes are transcriptionally induced by ABA treatment (Arabidopsis eFP Browser Database and Arabidopsis Hormone Database). Moreover, despite the fact that as many as 2140 *Arabidopsis* genes contain the motif in their promoter sequence (Supplementary Table S7 at *JXB* online), almost all of them are involved in the responses to various abiotic stimuli, including drought stress and ABA treatment. *SAG113*, which encodes a Golgi-localized, highly ABA-induced protein phosphatase 2C (PP2C; Zhang and Gan, 2012), is among the 61 genes containing the CACGTGT motif. *SAG113* is expressed in senescing leaves and its transcript levels are significantly reduced in the *aba2* and *abi4* ABA biosynthesis/signalling mutants. It has been shown that *SAG113* is a direct target gene of the NAC transcription factor AtNAP (also called ANAC029), a key regulator of leaf senescence, and that it is specifically involved in the control of water loss during leaf senescence (Zhang and Gan, 2012). Among the 61 genes containing the CACGTGT motif, nine encode transcription factors, five of which are ABA-induced NAC factors (*ANAC029/AtNAP*, *ANAC055*, *ANAC062*, *ANAC072/RD26*, and *ANAC102*).

GMCACGT is probably an extended version of the bZIP motif, since it encompasses the CACGT sequence. It has been identified with a higher *P*-value than the other motifs (*P*=0.01322) and, genome wide, 3251 genes were found to contain the motif in their promoter sequence (Supplementary Table S7 at *JXB* online). A total of 805 of these genes contain GMCACGTGT, representing a ‘merged’ variant of the above-reported CACGTGT G-box and GMCACGT, while 516 genes contain both of them in their promoters but as separate CREs.

Function enrichment analysis indicated that in addition to the functions identified for the CACGTGT element, GMCACGT is present in a range of genes related to sugar metabolism and protein transport. This finding supports the notion that GMCACGT might have a specific regulatory function, different from that of the G-box element. On the other hand, 805 genes containing the merged GMCACGTGT motif are highly enriched in genes involved in photosynthesis, including genes coding for proteins of the photosynthetic complexes.

Another significantly enriched motif (*P*=0.000131) in cluster 26 is AAGTCAA which was previously identified as a motif over-represented in clusters enriched in singlet oxygen-modulated genes (Petrov *et al.*, 2012). While there are as many as 5344 genes in the *Arabidopsis* genome containing the AAGTCAA motif (Supplementary Table S7 at *JXB* online), the enrichment analysis shows that these genes are associated with specific stress responses and metabolic functions. In contrast to genes containing the bZIP motif, the AAGTCAA motif is related rather to ‘biotic stimulus’, including ‘immune response’, ‘response to chitin’, and ‘programmed cell death’. Moreover, genes related to transmembrane receptor activity and a range of catalytic activities are also over-represented. Compartment-wise, the AAGTCAA motif is highly specific to genes encoding membrane proteins located in the endoplasmic reticulum and plasma membranes.

Another previously characterized motif is CCGCGT. Although in the present analysis no significant functional enrichment was found for the genes containing this CRE in their promoters, it has previously been reported that CCGCG belongs to the conserved DNA motifs (CMs) present in the promoters of the cold-responsive transcription factor genes *CBF2* and *ZAT12* (Vogel, 2005; Doherty *et al.*, 2009). The present experiments indicate a function for the motif in mediating salt stress-induced senescence and the response to oxidative stress.

The three remaining putative CREs did not exhibit significant enrichment in any functional gene category, which might be due to the fact that they occurred in only a limited number of genes (e.g. TCSTYGACG in only 218, and ACGCGGT in 372 of all *Arabidopsis* genes; Supplementary Table S7 at *JXB* online), or due to the fact that the genes putatively regulated via these CREs are not strictly defined by GO [here only functional categories exhibiting a false discovery rate (FDR) ≤0.05 are reported].

Summarizing, the analysis of the *cis*-elements involved in salt stress-induced senescence, developmental senescence, and the oxidative stress response resulted in the identification of seven *cis-*regulatory motifs, three of which are previously-characterized CREs involved in ROS signalling. Additionally, some of these CREs were found to be present in genes encoding proteins of related physiological functions and subcellular localization.

### Co-expression analysis

**Fig. 4.**
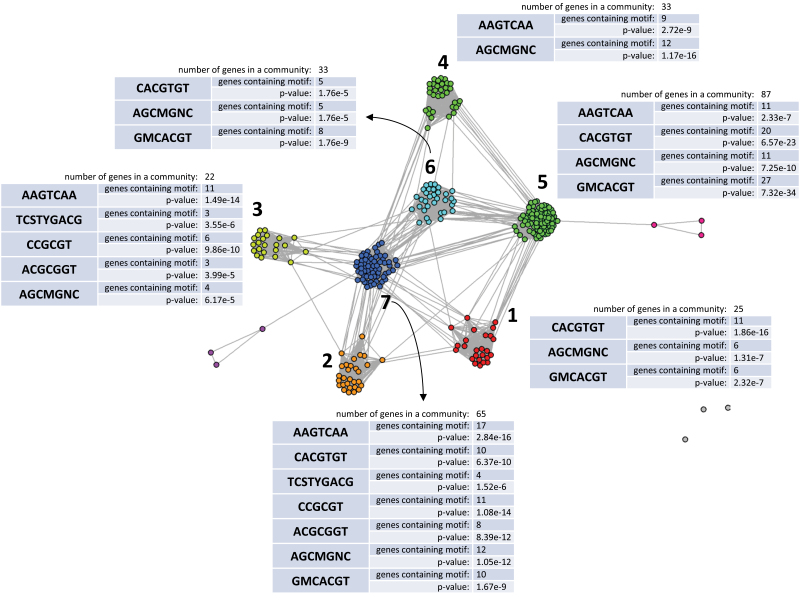
Network representation of the correlation structure of genes responsive to long-term moderate salt stress, oxidative stress, and affected during developmental senescence. All network communities are colour-coded; for communities containing more than five genes (1–7), the CREs enriched in the gene’s promoters (with a *P*-value cut-off of 0.0001) are listed.

In a second step, the question was asked of how many separate regulatory modules are found in cluster 26, defined as sets of genes exhibiting different temporal patterns of expression, and whether these modules are connected to the identified CREs. As initially stated, such an analysis was impossible using the original data set, mainly due to the low number of data points and large number of genes analysed. It was therefore decided to integrate other, publicly available data sets and to support the results with an extensive co-expression analysis. To address this task, an independent set of time series data, including salt stress, oxidative stress, and senescence, was used. For oxidative stress and salt stress responses, NASC arrays were used (experiments 143 and 140 from the AtGenExpress consortium), that include a 24h microarray time series experiment where samples were taken at 0.5, 1, 3, 6, 12, and 24h after treatment with salt (150mM NaCl) or 10 μM methyl viologen (paraquat) to induce oxidative stress (Kilian *et al.*, 2007). The senescence time series data were taken from Breeze *et al.* (2011) and included samples collected over 11 d of developmental leaf senescence (Supplementary Table S8 at *JXB* online). Although growth conditions and treatments in the time series experiments were different from those in the present experiment, as many as 85% of the genes identified as differentially expressed in the original data sets exhibit similar changes in the time series experiments (the same sign of the change and a *P*-value ≤0.01). The remaining 15% of the genes exhibited changes which were noisy and mostly of low magnitude in the time series experiments, and thus could not be classified statistically. This result shows that the response specificity in the time series experiments adequately resembles the present data despite the fact that, for example, methyl viologen instead of H_2_O_2_ was used to induce oxidative stress. The data were therefore used to reconstruct three co-expression networks (one each for developmental senescence, salinity-induced senescence, and oxidative stress) of 350 genes up-regulated in all three conditions (from cluster 26). Using a correlation coefficient threshold of 0.6, an intersection network was identified. In this network, nodes represent 350 genes of cluster 26. Each pair of nodes is connected with an edge, if the correlation coefficient between two genes exceeds 0.6 in all three experimental conditions. In the intersection network, communities were identified using a fast greedy search algorithm (Clauset *et al.*, 2004). Community, in the context of network analysis, is defined as a group of nodes that are more densely connected internally than with the rest of the network. Thus, in the present gene correlation network, communities correspond to regulatory modules: groups of tightly co-expressed genes, probably being co-regulated by the same transcriptional regulators (Segal *et al.*, 2003). Figure 4 shows a network representation of cluster 26. Seven regulatory modules containing more than five genes each were identified within the network by the community search algorithm (Supplementary Table S9 at *JXB* online).

Remarkably, genes connected by an edge in this network tend to share one or more of the selected candidate CREs, with a frequency well beyond that expected by chance (*P*-value estimated by a permutation test <0.001). Comparison of the gene co-expression with the distribution of the candidate CREs in the cluster 26 genes indicated that certain CREs are characteristic for certain modules (Table 2). The CACGTGT and GMCACGT motifs are found only in modules 1 and 5–7, and in all of them are very significantly enriched. CCGCGT, ACGCGGT, and TCSTYGACG are specific for modules 3 and 7; AGCMGNC is present in all modules except module 2. Finally, AAGTCAA is distributed between multiple modules, with a significant enrichment in modules 3, 4, 5, and 7. The reciprocal check of the candidate CREs was done using single modules as a query in MEME CRE, and a positive verification was obtained for all hits except of the least significant (Table 2).

**Table 2.** Analysis of the CRE enrichment presented in network communities The *P*-values shown were estimated by Fisher’s exact test comparing the number of genes identified by MEME as containing the specified CRE in relation to the community size and frequency of the CRE in all gene promoters.

		Community						
		1	2	3	4	5	6	7
AAGTCAA	Genes containing motif	4	4	11	9	11	4	17
	*P*-value	0.000815	0.00145	1.49E-14	2.72E-09	2.33E-07	0.063647	5.54E-14
CACGTGT	Genes containing motif	11	2	2	1	20	5	10
	*P*-value	1.86E-16	0.034632	0.020618	0.286934	6.57E-23	0.001796	1.20E-08
TCSTYGACG	Genes containing motif	1	0	3	0	0	1	4
	*P*-value	0.036911	1	3.55E-06	1	1	0.123278	4.92E-06
CCGCGT	Genes containing motif	0	0	6	3	4	2	11
	*P*-value	1	1	9.86E-10	0.000675	0.001089	0.077927	3.14E-13
ACGCGGT	Genes containing motif	0	0	3	1	1	1	8
	*P*-value	1	1	3.99E-05	0.099643	0.242691	0.242691	9.37E-11
AGCMGNC	Genes containing motif	6	3	4	12	11	5	12
	*P*-value	1.31E-07	0.003029	6.17E-05	1.17E-16	7.25E-10	0.001796	3.89E-11
GMCACGT	Genes containing motif	6	2	0	3	27	8	10
	*P*-value	2.32E-07	0.041106	1	0.005719	7.32E-34	4.59E-06	3.07E-08

## Discussion

Leaf senescence is triggered prematurely by various environmental cues such as, for example, salinity (Volkmar *et al.*, 1998; Munns, 2002; Munné-Bosch and Alegre, 2004). Although several studies have been conducted in the past to profile the transcriptomes of salt-stressed *Arabidopsis* plants and during developmental senescence, to the authors’ knowledge, so far no attempt has been undertaken to identify regulatory components allowing cross-talk between salt-induced and developmental senescence. Table 3 represents a brief summary of *Arabidopsis* salinity stress gene expression profiling experiments reported so far. As indicated, most of the earlier studies were performed under experimental conditions that limited the investigation of leaf senescence induced by salinity. For example, a short duration of salinity stress (a few hours) is a condition in which leaves do not display typical senescence characteristics. Similarly, transcript analyses of whole seedlings including roots are not appropriate to study molecular processes relevant for leaf senescence. In this study, an experimental condition was therefore employed under which salinity stress induces symptoms characteristic for developmental senescence. It was shown that long-term (4 d), moderate (150mM) NaCl treatment of hydroponically grown, 28-day-old *Arabidopsis* plants induces leaf senescence as measured by chlorophyll content, photosynthetic activity, and the expression level of developmental senescence marker genes. Microarray-based expression profiling revealed that 797 out of 1051 salinity-up-regulated genes (~76%), and 380 out of 551 down-regulated genes (~69%) are known SAGs, strongly suggesting that salt-induced senescence and developmental senescence share signalling pathways in *Arabidopsis*. Furthermore, the transcript levels of 138 ROS-responsive genes were up-regulated upon 4 d of NaCl treatment, indicating an elevated accumulation of ROS after long-term salt stress. ROS are proposed as major candidate signalling molecules involved in salt stress signal transduction (Gomez *et al.*, 2004; Munns and Tester, 2008; Rubio *et al.*, 2009; Hanqing *et al.*, 2010; Chen *et al.*, 2012). Although an excess accumulation of cellular ROS which are finally converted to H_2_O_2_ leads to an oxidative burst or cell death, maintaining their appropriate level is critical for mediating the acquisition of tolerance to stress as well as signal transduction of plant growth and development. It has been shown that an increased level of H_2_O_2_ is one of the earliest events in plants during senescence (Bhattacharjee, 2005; Zimmermann *et al.*, 2006). Timing and progression of senescence is tightly regulated through synergistic or antagonistic interactions between various signalling molecules such as sugars, nitrogen, hormones, and ROS. It has been shown that the coordinated interplay between the H_2_O_2_-scavenging enzymes catalase 2 (CAT2) and ascorbate peroxidase 1 (APX1) leads to a distinct increase in H_2_O_2_ at the time plants start to bolt and enter senescence (Ye *et al.*, 2000; Zimmermann *et al.*, 2006). Various delayed or accelerated leaf senescence mutants (such as *ore1*, *ore3*, *ore9*, *jub1*, *vitc*, and *crp5/old1*) exhibit an altered antioxidant status, further suggesting ROS (H_2_O_2_) as promising candidates acting as nodes for the cross-talk between developmental senescence and stress signalling pathways (Woo *et al.*, 2004; Pavet *et al.*, 2005; Jing *et al.*, 2008; Wu *et al.*, 2012). The transition from the response to stress to the induction of senescence may occur as a result of a tight interaction between ROS and hormonal signalling networks, thereby allowing plants to regulate senescence under unfavourable environmental conditions. However, the regulatory role of ROS for the control of stress-induced senescence is currently not particularly clear. The identification of elements regulating SAGs through an involvement of ROS will therefore lead to significant progress in the understanding of stress-induced senescence.

**Table 3.** Summary of *Arabidopsis* salt stress-related gene expression profiling experiments Sample and treatment descriptions together with other relevant experimental parameters are listed.

NaCl concentration	Time points	Plant age	Tissue	Growth condition	Array format	Source	Remarks
200 mM	1, 12 h	10 d	Whole seedlings	Grown on solid medium transferred to liquid medium	Affymetrix whole- genome tiling array	1	High NaCl concentration, roots included
250 mM	2, 10 h	3 weeks	Whole seedlings	Treatment in hydroponic condition	Affymetrix whole- genome tiling array	2	High NaCl concentration, roots included
150 mM	3, 24 h	4 weeks	Plants	Whole plants	25 425, 70-mer oligonucleotide array	3	Short term
250 mM	2 h	2 weeks	Whole seedlings	Hydroponics	~7000 cDNA glass slide microarray	4	High NaCl concentration, roots included, low transcript coverage
100 mM	3, 27 h	4 weeks	Leaves, roots	Hydroponics	~8100 gene oligonucleotide chip	5	Low transcript coverage
250 mM	1, 2, 5, 10, 24 h	3 weeks	Whole plants	Hydroponics	~7000 cDNA glass slide microarray	6	High NaCl concentration, low transcript coverage

Data were taken from the following publications (‘Source’): 1, Zeller *et al.* (2009); 2, Matsui *et al.* (2008); 3, Gong *et al.* (2005); 4, Taji *et al.* (2004); 5, Kreps *et al.* (2002); 6, Seki *et al.* (2002).

All the above facts triggered an interest to study the participation of H_2_O_2_ in integrating regulatory networks of salinity- and age-triggered leaf senescence

**Fig. 5.**
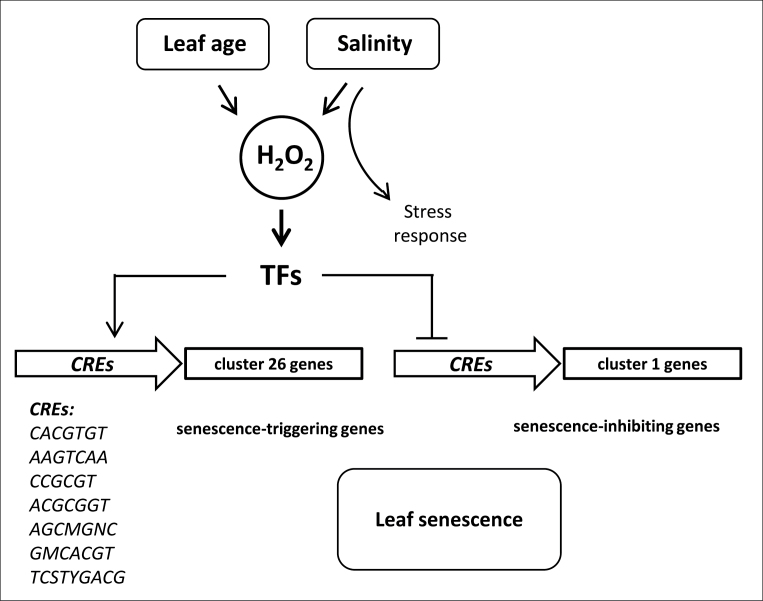
Model for the integration of H_2_O_2_ in developmental and salinity-induced senescence. Hydrogen peroxide (H_2_O_2_) accumulates in leaves during developmental senescence, as reported, for example, by Zimmermann *et al.* (2006). Salinity stress similarly triggers a rise in cellular H_2_O_2_ level. Transcription factors (TFs) responding to an elevated H_2_O_2_ level activate genes included in cluster 26, most probably by binding to *cis*-regulatory elements (CREs) identified here and by Petrov *et al.* (2012). Genes contained in cluster 1 may be down-regulated by TFs through unknown CREs. Salinity may additionally elicit stress responses not directly linked to senescence.

Genes whose expression is regulated by a common upstream transcription factor generally exhibit significant co-expression in conditions where the transcription factor is active. This has been shown for multiple regulons, such as those involved in the response to changing environments, nutrient availability, or being active during morphogenesis (Stuart *et al.*, 2003; Ma and Bohnert, 2007). This general characteristic allowed study of the properties of regulatory networks in *A. thaliana* at the genome scale; for example, by integrating data of 963 microarray chips from a wide range of experimental conditions, Mentzen and Wurtele (2008) estimated 998 regulons for *Arabidopsis*, ranging in size from one to 1623 genes. More importantly, however, besides giving a general overview of the regulatory network organization, the co-expression analysis was successfully applied to uncover regulatory modules having specific molecular functions. Examples include the co-expression analysis of enzymatic genes involved in indole, flavonoid, and phenylpropanoid biosynthetic pathways (Gachon *et al.*, 2005), the identification of new genes involved in the biosynthesis of cellulose and cell wall components (Brown *et al.*, 2005; Persson *et al.*, 2005), uncovering of a clade of brassinosteroid-related genes (Lisso *et al.*, 2005), and many others (reviewed by Usadel *et al.*, 2009). Co-regulation of multiple genes by a common upstream TF is related to the presence of a common CRE in their promoters (Dare *et al.*, 2008; Spitz and Furlong, 2012; Ma *et al.*, 2013). Therefore, the integration of efficient motif search algorithms (reviewed by Das and Dai, 2007) allowed the successful use of co-expression analysis in revealing the functions of certain transcription factors or regulatory elements. Examples of such approaches include Myb transcription factors regulating glucosinolate metabolism in *Arabidopsis* (Hirai *et al.*, 2007), the identification of targets of the OBP1 transcription factor in drought stress (Vandepoele *et al.*, 2009), the characterization of regulatory networks of WRKY (Berri *et al.*, 2009) and bZIP proteins (Wei *et al.*, 2012), transcriptional regulators involved in phytohormone signal transduction (van Verk *et al*., 2011), and many others (computational methods reviewed by Stifanelli *et al.*, 2013). All these studies showed that a combination of carefully designed co-expression analyses (including proper choice of experiments, data type, and computational strategy) with a potent motif search algorithm is a powerful approach for the identification of new condition-specific regulons and new transcription factor targets. In this study, the transcript profiles of salt-induced senescence, developmental senescence, and H_2_O_2_-treated samples were compared. Clustering of microarray expression profiles for all genes differentially expressed during developmental senescence, salt-induced senescence, and treatment with H_2_O_2_ resulted in 26 possible patterns of expression. Promoter CRE analysis was performed only on clusters containing genes with similar expression behaviour among all three experimental conditions (i.e. clusters 1 and 26). The present data suggest that long-term moderate salt stress, H_2_O_2_ signalling, and senescence trigger a common transcriptional programme, characterized by several tightly co-regulated gene clusters sharing specific CREs (Fig. 5). Co-expression-based analysis of the *cis-*elements involved in the H_2_O_2_ response as well as salt-induced and developmental senescence resulted in the identification of three previously characterized CREs involved in stress signalling (CACGTGT, AAGTCAA, and CCGCGT), indicating the reliability of the computational analysis pipeline. Additionally, four new putative CREs, namely ACGCGGT, AGCMGNC, GMCACGT, and TCSTYGACG, were identified, one of which (GMCACGT) was enriched in promoters of genes involved in specific biological processes (Supplementary Table S6 at *JXB* online), thus probably playing a role in shaping the transcriptional response to the applied conditions. The CACGTGT is an extended CACGTG motif, one of the most common palindromic G-boxes/ABREs (abscisic acid response elements), also known as a bZIP-binding motif (Jakoby *et al.*, 2002; Toledo-Ortiz *et al.*, 2003; Chintalapati and Rajendra, 2004). The bZIP transcription factors interact as dimers with ABREs, which are ACGT-containing ‘G-boxes’ in promoters (Hattori *et al.*, 2002). G-boxes are involved in the response to abiotic stress (anaerobiosis, cold, ultraviolet light irradiation) and hormone signalling, especially ABA (Menkens *et al.*, 1995; Shinozaki and Yamaguchi-Shinozaki, 1997; Gilmour *et al.*, 1998). ABA is a key hormone for the regulation of plant growth, development, and stress adaptation (Davies and Jones, 1991; Giraudat *et al.*, 1994; Finkelstein *et al.*, 2002; Chaves *et al.*, 2009; Ashraf, 2010; Xue-Xuan *et al.*, 2010). ABA is also known as a hormone triggering senescence, and some *Arabidopsis* mutants with deficiencies in ABA biosynthesis or signalling have been reported to exhibit a changed senescence programme (Pourtau *et al.*, 2004; Lim and Nam, 2005; Passioura, 2007; Yang *et al.*, 2011). Various studies suggest interplay between ROS and ABA signalling, implicating ROS as second messengers in ABA signal transduction pathways (Pei *et al.*, 2000; Joo *et al.*, 2001; Murata *et al.*, 2001; Torres *et al*., 2002, 2005; reviewed by Cho *et al.*, 2009). For example, cellular ROS levels are enhanced by ABA treatment in *Arabidopsis* guard cells (Pei *et al.*, 2000). Furthermore, ABA increases H_2_O_2_ levels in maize embryos and seedlings, and in *Vicia faba* guard cells, a process that precedes stomatal closure (Guan *et al.*, 2000; Zhang *et al.*, 2001; Jiang and Zhang, 2002, 2003). The enrichment of CACGTGT in cluster 26 genes suggests it as a core stress-specific CRE linking salt and oxidative stress responses with senescence. Additionally, one of the new putative CREs, GMCACGT, exhibits a high sequence overlap with the bZIP element. In accordance with the present findings, Petrov *et al.* (2012) recently reported the G-box/ABRE-containing element GACACGTG to be over-represented in promoters of genes responding to more than one type of ROS (singlet oxygen-up-regulated genes, superoxide-up- and down-regulated genes, and H_2_O_2_-down-regulated genes).

Another previously reported CRE, namely AAGTCAA, was identified by Petrov *et al.* (2012) as being enriched among singlet oxygen-modulated genes, although no further characterization of the CRE was reported. Its relatively high frequency (presence in almost 2000 *Arabidopsis* genes), however, suggests that it plays a role in mediating more general stress signals or that it acts as an enhancer coupled with more specific binding sites. Remarkably, genes containing the bZIP element and AAGTCAA differ significantly in terms of their function. Whereas genes containing different forms of the bZIP element are involved in abiotic stress responses and signalling, the AAGTCAA element is found in genes involved in biotic stress defence and a range of metabolic processes. Additionally, the extended bZIP element GMCACGTGT appears to be present in genes encoding photosynthesis-related proteins. Thus, an emerging picture of gene expression regulation in response to salt stress and oxidative stress, and during senescence suggests that at least these two CREs are common for all of these conditions, while the other five reported might have auxiliary roles.

In summary, the present analysis further supports the model that developmental and salt-triggered senescence share H_2_O_2_ signalling pathways in *Arabidopsis*. In this study, three previously characterized and four novel putative CREs probably involved in the response to H_2_O_2_ treatment and in both types of senescence, and thus representing potential regulatory elements acting at cross-talk points of the three physiological processes, were identified. Six of the seven identified motifs are significantly enriched in genes sharing specific molecular functions. In addition, the highly specific associations of individual motifs with certain functional gene categories reflect a hierarchical and function-specific organization of the transcription regulatory network. Future work should be directed towards understanding the biological relevance of the newly identified motifs *in planta*.

## Supplementary data

Supplementary data are avasilable at *JXB* online.

Table S1. Expression of ROS-responsive genes determined by qRT-PCR.

Table S2. Genes differentially expressed after 4 d of salinity stress.

Table S3. Comparison of genes differentially expressed during salt-induced senescence, developmental senescence, and upon H_2_O_2_ treatment.

Table S4. Transcripts responsive to 1h and 5h H_2_O_2_ (10mM) treatment.

Table S5. Classification of gene response specificity and number of genes in clusters.

Table S6. Function enrichment analysis of genes containing identified *cis*-regulatory elements.

Table S7. Genes of the *Arabidopsis* genome containing each CRE described in the manuscript.

Table S8. AtGenExpress data sets.

Table S9. Classification of the genes according to their response to individual treatments (gene cluster) and their co-expression with genes belonging to the same cluster (gene community).

Supplementary Data
