# Supplementary material for: Salt stress and senescence: identification of cross-talk regulatory components
Source: J Exp Bot. 2014 May 6;65(14):3993–4008. doi: 10.1093/jxb/eru173 (PMC4106443; doi:10.1093/jxb/eru173)
Supplement: Supplementary Data [file supp_65_14_3993__index.html]

Salt stress and senescence: identification of cross-talk regulatory components — Salt stress and senescence: identification of cross-talk regulatory components — Supplementary Data 

# Salt stress and senescence: identification of cross-talk regulatory components

## Supplementary Data

Data files

**Files in this Data Supplement:**

- Supplementary Data - Supplementary Data
